# Transition metal ions and their oxide nanoparticles differentially regulate CHO cell growth, metabolism, and N-glycosylation

**DOI:** 10.3389/fbioe.2026.1978281

**Published:** 2026-10-28

**Authors:** Chao Yu, Xuejing Yao, Zhenglong Tian, Yi Gao, Shilong Wang, Cuicui Jiang, Qisheng Liu

**Affiliations:** 1 School of Life Science and Technology, Tongji University, Shanghai, China; 2 Shanghai SPH Jiaolian Pharmaceutical Technology Co., Ltd., Shanghai, China; 3 Research Center for Translational Medicine at East Hospital, School of Life Sciences and Technology, Tongji University, Shanghai, China; 4 Cathay Biotech Inc., Shanghai, China; 5 Yantai Zhenghai Bio-Tech Co., Ltd., Yantai, China

**Keywords:** cell metabolism, Chinese hamster ovary cells, MnFe-LDH, nanoparticles, N-glycosylation, transcriptomics

## Abstract

Trace metal ions act as essential cofactors but exhibit concentration-dependent cytotoxicity that shapes Chinese hamster ovary (CHO) cell culture performance. This work systematically evaluated six transition metal ions (Fe^3+^, Mn^2+^, Cu^2+^, Zn^2+^, Co^2+^, Ni^2+^) and their metal oxide nanoparticles for their impacts on cell proliferation, metabolism and protein N-glycosylation. Single-metal screening revealed dose-dependent effects: low Fe^3+^ and Mn^2+^ boosted cell growth, while high Cu^2+^ and Zn^2+^ triggered severe cytotoxicity. Nanoparticles displayed milder toxicity than ionic metals owing to slow dissolution kinetics. Moderate combined supplementation of Mn^2+^/Fe^3+^ ions or as-synthesized layered double hydroxide (LDH) nanoparticles (MnFe-LDH) optimized 14-day batch culture, improving cell viability, protein yield and N-glycan galactosylation. Transcriptomic data identified mitochondrial respiratory chain as the primary responsive pathway, with six subunits of complexes I–V (COX2, COX3, ATP6, ND3, ND4, ND4L) upregulated in a dose-dependent fashion. This study offers mechanistic support for rational trace metal feeding strategies in CHO-based biopharmaceutical production, although practical translation of nanoparticle-based supplementation will additionally require control of particle dissolution behavior and removal of nanoparticle residues during downstream processing.

## Introduction

Monoclonal antibodies (mAbs) and other recombinant glycoproteins produced by Chinese hamster ovary (CHO) cells represent the dominant class of biotherapeutics in the pharmaceutical industry today (Walsh, 2018; Lalonde and Durocher, 2017). These complex proteins undergo critical post-translational modifications, particularly N-linked glycosylation, that significantly influence their stability, immunogenicity, pharmacokinetics, and clinical efficacy (Jimenez del Val et al., 2011). The glycosylation profile of therapeutic proteins is therefore considered a critical quality attribute (CQA) that must be tightly controlled during biomanufacturing (Gyorgypal et al., 2025). Cell culture media composition affects not only cell growth and productivity but also the glycosylation patterns of the expressed proteins, yet achieving consistent product quality remains challenging despite decades of process development (Weiss et al., 2021; Weiss et al., 2022; Polanco et al., 2023). Trace metals are among the most consequential media components because they act as essential enzymatic cofactors at physiological concentrations but can become toxicants when present in excess (Jomova et al., 2022).

The genomic characterization of CHO cells has revealed extensive genetic heterogeneity across different cell lines, which contributes to variable responses to culture conditions (Xu et al., 2011; Lewis et al., 2013). This diversity complicates the development of universal media formulations; cell line engineering and media optimization have therefore become central, complementary strategies for controlling product quality in CHO-based manufacturing (Fischer et al., 2015). Metal oxide nanoparticles have emerged as potential vehicles for trace metal supplementation because they exhibit slower dissolution kinetics that may provide sustained ion release while reducing acute cytotoxicity (Hanot et al., 2015; Chusuei et al., 2013). Hanot et al. showed that iron-oxide nanoparticle surface chemistry profoundly influences uptake kinetics and cytotoxicity in CHO-K1 cells, and that cellular uptake occurs predominantly through endocytosis into acidic endolysosomal compartments (pH 4.5–5.0), where gradual dissolution and metal ion release take place (Hanot et al., 2015; Wang et al., 2013).

Manganese has received particular attention due to its role as an essential cofactor for β-1,4-galactosyltransferase (B4GALT1), the enzyme responsible for terminal galactosylation of N-glycans (Kildegaard et al., 2016; Ramakrishnan and Qasba, 2002), and genetic engineering of B4GALT1 expression has been used to optimize antibody galactosylation in CHO cells (Umaña et al., 1999). Previous studies have shown that manganese supplementation enhances mAb galactosylation in a dose-dependent manner (Gyorgypal et al., 2025; Gramer et al., 2011; Williamson et al., 2018), while the influence of terminal galactosylation on antibody effector functions relative to core fucosylation remains debated (Fe et al., 2011; Shinkawa et al., 2003). Understanding these metal-glycosylation relationships is essential for rational media design.

This study systematically evaluates the effects of six transition metal ions (Fe^3+^, Mn^2+^, Cu^2+^, Zn^2+^, Co^2+^, Ni^2+^) and their corresponding oxide nanoparticles on CHO cell growth, metabolism, and N-glycosylation. Individual and combined metal treatments are investigated to identify optimal concentration ranges and assess whether nanoparticle-mediated delivery offers advantages over traditional ionic supplementation, and the molecular mechanisms underlying metal-induced changes are further examined through transcriptomic analysis, with a focus on mitochondrial respiratory chain components and glycosylation pathway enzymes.

## Materials and methods

### Cell culture and metal treatments

Chinese hamster ovary (CHO) cells were cultured in MaxP CHO 100 medium (Thermo Fisher Scientific, Waltham, MA, United States) at 37 °C in a humidified atmosphere containing 8% CO_2_ with orbital shaking at 300 rpm and 80% relative humidity. Cells were seeded at an initial density of 1.5 × 10^6^ cells/mL in 20 mL working volume for batch culture experiments. For single-metal screening, six metal ions (Fe^3+^, Cu^2+^, Zn^2+^, Mn^2+^, Co^2+^, Ni^2+^) and their corresponding metal oxide nanoparticles (Fe_2_O_3_, CuO, ZnO, MnO_2_, Co_3_O_4_, NiO) were added to the culture medium at serially diluted concentrations, with an untreated blank Control group as reference. Metal salts (FeCl_3_.6H_2_O, CuSO_4_.5H_2_O, ZnSO_4_.7H_2_O, MnCl_2_.4H_2_O, CoCl_2_.6H_2_O, NiCl_2_) were obtained from Sigma-Aldrich (St. Louis, MO, United States), and metal oxide nanoparticles (purity >99.5%) were purchased from Aladdin Biochemical Technology (Shanghai, China). For the synthesis of Fe-Mn layered double hydroxide (MnFe-LDH), 0.48 g Mn(NO_3_)_2_·4H_2_O, 0.111 g Fe(NO_3_)_3_·9H_2_O and 600 mg urea were dissolved in 40 mL double-distilled water. The resulting mixture was stirred vigorously for 30 min to yield a clear homogeneous solution, which was then transferred to a Teflon-lined stainless steel autoclave and subjected to hydrothermal treatment at 180 °C for 24 h. After the reaction, the precipitate was collected via centrifugation at 20,000 rcf, rinsed thoroughly with deionized water three times, and stored at 4 °C for subsequent use. Each treatment was performed in triplicate and maintained for 6–8 days with daily sampling. Nanoparticle stock suspensions were prepared by ultrasonic dispersion in sterile deionized water for 30 min before addition to culture medium. All chemicals were of analytical grade and used without further purification. Based on single-metal screening results, combined treatments of Mn^2+^+Fe^3+^ ion mixtures and MnFe-LDH nanoparticle composites were applied at four concentration gradients (designated A (0.01 mM Mn^2+^ and 0.002 mM Fe^3+^), B (0.05 mM Mn^2+^ and 0.012 mM Fe^3+^), C (0.1 mM Mn^2+^ and 0.025 mM Fe^3+^), D (0.2 mM Mn^2+^ and 0.049 mM Fe^3+^) from low to high) for extended 14-day batch culture experiments.

### Metal and nanoparticle treatment concentrations

For single-ion treatments, stock solutions of FeCl_3_.6H_2_O (10 mM Fe^3+^), CuSO_4_.5H_2_O (10 mM Cu^2+^), ZnSO_4_.7H_2_O (10 mM Zn^2+^), MnCl_2_.4H_2_O (10 g/L Mn^2+^), CoCl_2_.6H_2_O (10 g/L Co^2+^), and NiCl_2_ (2.8 M Ni^2+^, prepared by dissolving NiO powder in 6 M HCl) were added to the culture medium at the following concentrations: Fe^3+^ at 0.18, 0.36, 0.72, and 1.44 mM; Cu^2+^ at 0.12, 0.23, 0.46, and 0.92 mM; Zn^2+^ at 0.004, 0.02, 0.04, and 0.1 mM; Mn^2+^ at 0.1, 0.25, 0.5, and 1.0 mM; Co^2+^ at 0.02, 0.05, 0.1, and 0.2 mM; and Ni^2+^ at 0.2, 0.5, 1.0, and 2.0 mM.

For single-metal oxide nanoparticle treatments, powdered Fe_2_O_3_ was weighed directly into culture medium at 0.18, 0.36, 0.72, 1.44, 2.86, and 5.73 mM; CuO at 0.12, 0.23, 0.46, and 0.92 mM; ZnO at 0.02, 0.04, 0.08, 0.1, 0.22, and 0.43 mM; MnO_2_ at 0.25, 0.5, and 1.0 mM; Co_3_O_4_ at 0.02, 0.05, 0.1, and 0.2 mM; and NiO at 0.5, 1.0, and 2.0 mM. Nanoparticle sizes were 18.62 nm (Fe_2_O_3_), 20 nm (CuO), 34.24 nm (ZnO), 100–150 nm (MnO_2_), 32.81 nm (Co_3_O_4_), and 4.5 nm (NiO) as characterized by the manufacturer.

For combined Fe/Mn ion treatments, a mixed stock solution containing MnCl_2_.4H_2_O (10 g/L, ∼50.53 mM) and FeCl_3_.6H_2_O (2.65 g/L, ∼10 mM) was prepared. Four experimental groups (A, B, C, D) with increasing Mn^2+^ and Fe^3+^ concentrations were established in triplicate. For combined MnFe-LDH nanoparticle treatments, four groups (A, B, C, D) with increasing nanoparticle concentrations were similarly established. Gradient levels were anchored to the single-metal screening results: gradient A corresponded to the lowest level that did not impair cell growth, gradients B and C fell within the moderate range that sustained near-normal proliferation and metabolism, and gradient D exceeded the high-dose toxicity threshold identified in the corresponding single-metal treatments.

### TEM, XRD and DLS characterization of MnFe-LDH

For TEM and HRTEM analysis, the MnFe-LDH powder was dispersed in deionized water by ultrasonication for 10 min, and a drop of the resulting suspension was deposited onto a carbon-coated copper microgrid and dried at room temperature before examination. The morphology, lateral dimensions and lattice fringes of the nanosheets were examined on a JEM-2100F transmission electron microscope (JEOL Ltd., Tokyo, Japan) operated at 200 kV in bright-field and high-resolution modes, and digital images were acquired with an EMSIS Quemesa camera (EMSIS GmbH, Muenster, Germany) at nominal magnifications of 200,000 x, 600,000 x, 800,000 x and 1,500,000 x.

The crystal phase of the as-prepared MnFe-LDH powder was examined by powder X-ray diffraction (XRD; Rigaku SmartLab SE, Rigaku Corporation, Tokyo, Japan) with Cu Kα radiation (λ = 1.5406 Å) over a 2θ range of 10°–80° at a continuous scanning rate of 2°/min. The diffraction pattern of the as-prepared powder was recorded as raw intensity versus 2θ without background subtraction. The hydrodynamic size distribution of MnFe-LDH was determined by dynamic light scattering (DLS) on a Zetasizer Nano ZS90 (Malvern Panalytical, Malvern, United Kingdom). For DLS, the nanoparticle powder was dispersed in sterile deionized water by ultrasonication for 30 min and passed through a 0.22 μm membrane filter before measurement, and volume-weighted size distributions were recorded in three replicate measurements. Measurements were performed at room temperature, and intensity data were collected at a fixed scattering angle of 90°; volume-weighted distributions were derived from the intensity distributions using the instrument software.

### Viable cell density and viability assays

Viable cell density (VCD) and cell viability were determined daily using the trypan blue exclusion method with an automated cell counter (Countess II FL, Thermo Fisher Scientific, United States). Briefly, 10 μL of cell suspension was mixed with 10 μL of 0.4% trypan blue solution, loaded onto a disposable hemocytometer slide, and analyzed according to the manufacturer’s protocol. Cell viability was calculated as: Viability (%) = (live cells/total cells) × 100.

### Metabolite analysis

Culture supernatants were collected daily by centrifugation at 1,000 × g for 5 min at 4 °C to remove cells and debris. Glucose, glutamic acid, and lactic acid concentrations were quantified using commercially available enzymatic assay kits according to the manufacturers’ instructions. Glucose was measured using a glucose oxidase-peroxidase (GOx-POD) based kit (Sigma-Aldrich). The enzymatic reaction produces a colored product proportional to glucose concentration, which was measured spectrophotometrically at 540 nm. A standard curve was generated using D-glucose standards (0–10 mM). Lactic acid concentration was determined using a lactate dehydrogenase (LDH)-based assay kit (Sigma-Aldrich). In alkaline conditions (pH 8.9), lactate is oxidized to pyruvate by LDH in the presence of NAD^+^, generating NADH which was measured at 340 nm. Glutamic acid was quantified using a glutamate dehydrogenase (GDH)-based assay (Sigma-Aldrich). Glutamate is deaminated by GDH in the presence of NAD^+^, producing α-ketoglutarate and NADH, which was measured at 340 nm. All measurements were performed in triplicate using a microplate reader (BioTek Synergy H1, Agilent Technologies, United States).

### Total protein quantification

Total secreted protein concentration in culture supernatant was determined on days 12, 13, and 14 of the extended batch culture using the bicinchoninic acid (BCA) protein assay kit (Pierce™ BCA Protein Assay Kit, Thermo Fisher Scientific). Briefly, 25 μL of sample or BSA standard (0–2000 μg/mL) was mixed with 200 μL of BCA working reagent (50:1 reagent A:B) and incubated at 37 °C for 30 min. Absorbance was measured at 562 nm using a microplate reader. A standard curve was generated from BSA standards, and sample protein concentrations were calculated by interpolation.

### N-glycan profiling

N-linked glycans were analyzed from culture supernatant collected on day 13. Proteins were denatured by heating at 95 °C for 5 min in the presence of SDS and dithiothreitol (DTT). N-glycans were enzymatically released by incubation with peptide-N-glycosidase F (PNGase F; New England Biolabs) at 37 °C overnight according to the manufacturer’s protocol. Released glycans were purified using hydrophilic interaction liquid chromatography (HILIC) solid-phase extraction and labeled with 2-aminobenzamide (2-AB) by reductive amination at 65 °C for 2 h. Labeled glycans were separated by HILIC-ultra performance liquid chromatography (HILIC-UPLC) on an Acquity UPLC system equipped with a BEH Amide column (2.1 × 150 mm, 1.7 μm; Waters Corporation, United States) and detected by fluorescence (excitation 330 nm, emission 420 nm). Glycan structures were identified by comparison with a glucose homopolymer standard ladder (GU values) and expressed as relative percentages of total glycan peak area. Major glycan subtypes (high-mannose, hybrid, and complex-type glycans) were classified based on structural characteristics.

### Transcriptome sequencing and analysis

Total RNA was extracted from cell pellets using TRIzol reagent (Invitrogen) following the manufacturer’s instructions. RNA integrity was assessed using an Agilent Bioanalyzer 2,100 (Agilent Technologies, United States) with RNA Integrity Number (RIN) ≥ 7.0 required for library construction. RNA sequencing libraries were prepared using the NEBNext Ultra II RNA Library Prep Kit (New England Biolabs) and sequenced on an Illumina NovaSeq 6,000 platform to generate 150 bp paired-end reads. Raw reads were quality-filtered using Trimmomatic (v0.39) with parameters: LEADING:3, TRAILING:3, SLIDINGWINDOW:4:15, MINLEN:36. Clean reads were aligned to the *Cricetulus griseus* reference genome (NCBI Assembly: GCF_000223,135.1) using STAR (v2.7.10a) with default parameters.

Gene expression levels were quantified as Transcripts Per Million (TPM) using featureCounts (v2.0.1). Differentially expressed genes (DEGs) were identified using DESeq2 (v1.38.3) with thresholds of |log_2_ (fold change)| ≥ 1 and adjusted P-value <0.05. DEGs were annotated and classified using Cluster of Orthologous Groups (COG) database. Gene Ontology (GO) enrichment analysis was performed using the clusterProfiler R package (v4.6.2) with Benjamini-Hochberg correction for multiple testing.

Expression levels of six core mitochondrial respiratory chain genes (COX2, COX3, ATP6, ND3, ND4, ND4L, B4GALT1) were extracted from transcriptome data and expressed as log_10_ (TPM+1) for statistical comparison. One-way analysis of variance (ANOVA) followed by Tukey’s honestly significant difference (HSD) post-hoc test was performed to evaluate significant differences among all treatment groups using GraphPad Prism 9 (GraphPad Software, United States). Statistical significance was defined as P < 0.05.

### Statistical analysis

All experiments were performed in triplicate unless otherwise stated. Data are presented as mean ± standard deviation (SD). For VCD, viability, and metabolite time-course data, statistical comparisons between treatment groups and Control at each time point were performed using two-way ANOVA with Bonferroni correction. For N-glycan distribution and gene expression analysis, one-way ANOVA with Tukey’s multiple comparison test was applied. Differences were considered statistically significant at P < 0.05. All statistical analyses were conducted using GraphPad Prism 9 (GraphPad Software, United States) or R statistical software.

## Results

### Single metal ion and metal oxide nanoparticle treatments modulate cell proliferation, viability and central carbon metabolism

To systematically characterize the independent biological effects of six common metal elements and their corresponding metal oxide nanoparticles on cultured cells, we subjected cells to serially diluted gradients of Fe^3+^/Fe_2_O_3_, Cu^2+^/CuO, Zn^2+^/ZnO, Mn^2+^/MnO2, Co^2+^/Co_3_O_4_ and Ni^2+^/NiO, with an untreated blank Control group as the reference. Viable cell density (VCD), cell viability and key metabolic substrates in culture supernatant were monitored daily across 6–8 days of continuous incubation (Figures 1–5). For all metal element series, low-to-moderate concentrations of metal ions and nanoparticles exerted mild promoting effects on cell proliferation, with VCD slightly elevated relative to the Control group during the mid-logarithmic growth phase. In contrast, high-dose treatments induced severe growth suppression, accompanied by a pronounced decline in cell viability (Figures 1, 2). Notably, metal oxide nanoparticles generally exhibited weaker cytotoxicity at equivalent molar concentrations compared to their soluble metal ion counterparts, consistent across all six metal systems. Dynamic profiles of primary carbon metabolites revealed coordinated shifts in glucose consumption and byproduct accumulation under metal stress (Figures 3–5). Residual glucose declined rapidly in all groups during active cell proliferation, whereas high-dose metal exposure slowed glucose catabolism and extended glucose retention in the medium (Figure 3). Lactic acid, the primary glycolytic waste product, accumulated progressively in low-concentration groups but exhibited suppressed production and accelerated clearance under toxic high-dose metal treatments (Figure 5). Glutamic acid, a critical nitrogen and carbon intermediate, displayed element-specific dynamic fluctuations; treatments with Mn, Co. and Ni compounds induced more dramatic perturbations in glutamic acid homeostasis relative to Fe, Cu and Zn series (Figure 4). Collectively, these single-metal screening results confirmed dose-dependent dual effects of metal species on cell growth and core energy metabolism, and identified Mn and Fe as candidate elements for subsequent combined treatment investigation.

**FIGURE 1:**
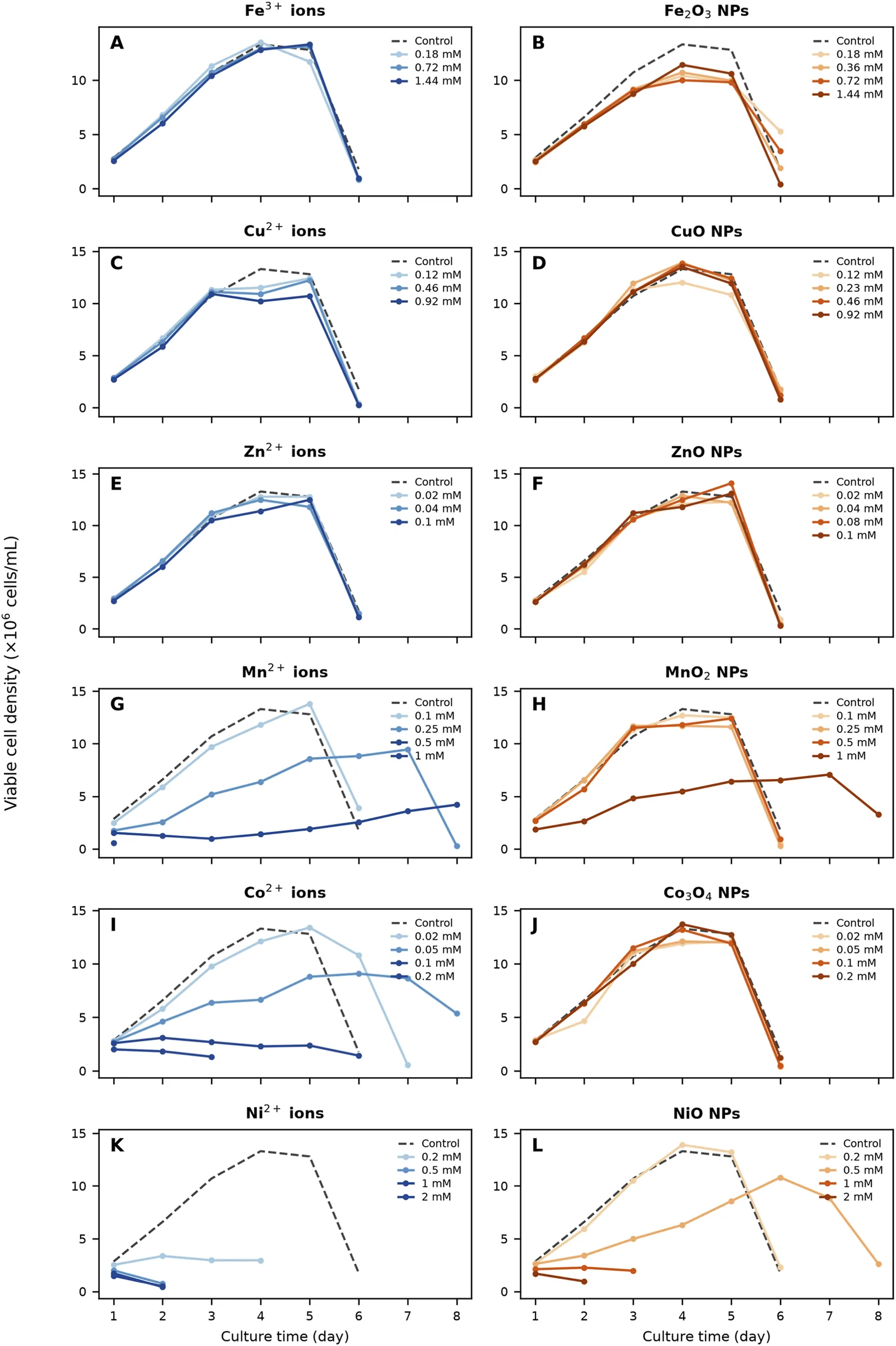
Dynamic viable cell density curves over incubation time for cells exposed to gradient concentrations of metal ions and metal oxide nanoparticles. **(A,B)** Fe^3+^ ions and Fe_2_O_3_ NPs; **(C,D)** Cu^2+^ ions and CuO NPs; **(E,F)** Zn^2+^ ions and ZnO NPs; **(G,H)** Mn^2+^ ions and MnO_2_ NPs; **(I,J)** Co^2+^ ions and Co_3_O_4_ NPs; (K,L) Ni^2+^ ions and NiO NPs.

**FIGURE 2:**
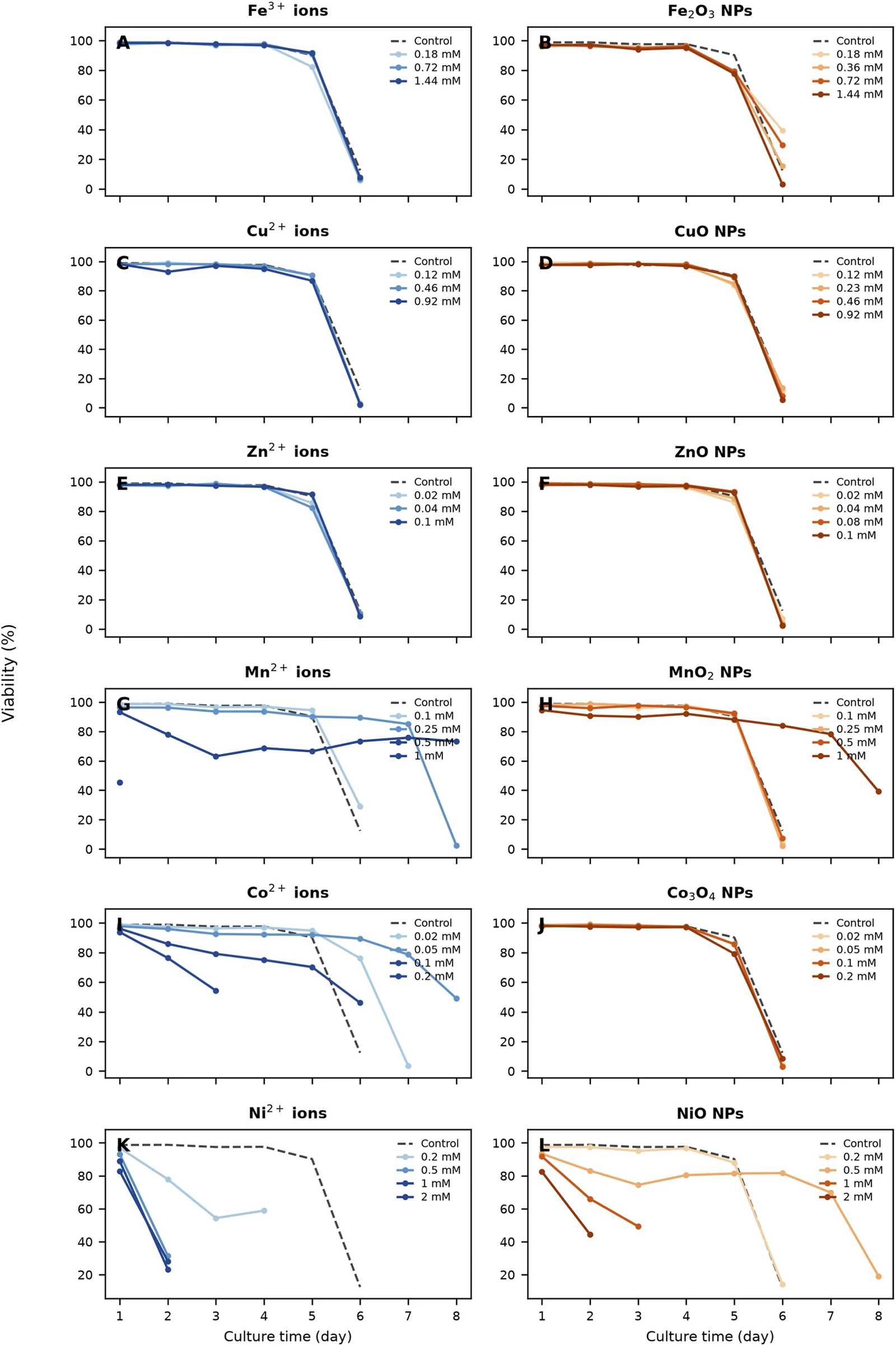
Dynamic cell viability curves over incubation time for cells exposed to serially diluted metal ions and metal oxide nanoparticles. **(A,B)** Fe^3+^ and Fe_2_O_3_ NPs; **(C,D)** Cu^2+^ and CuO NPs; **(E,F)** Zn^2+^ and ZnO NPs; **(G,H)** Mn^2+^ and MnO_2_ NPs; **(I,J)** Co^2+^ and Co_3_O_4_ NPs; **(K,L)** Ni^2+^ and NiO NPs.

**FIGURE 3:**
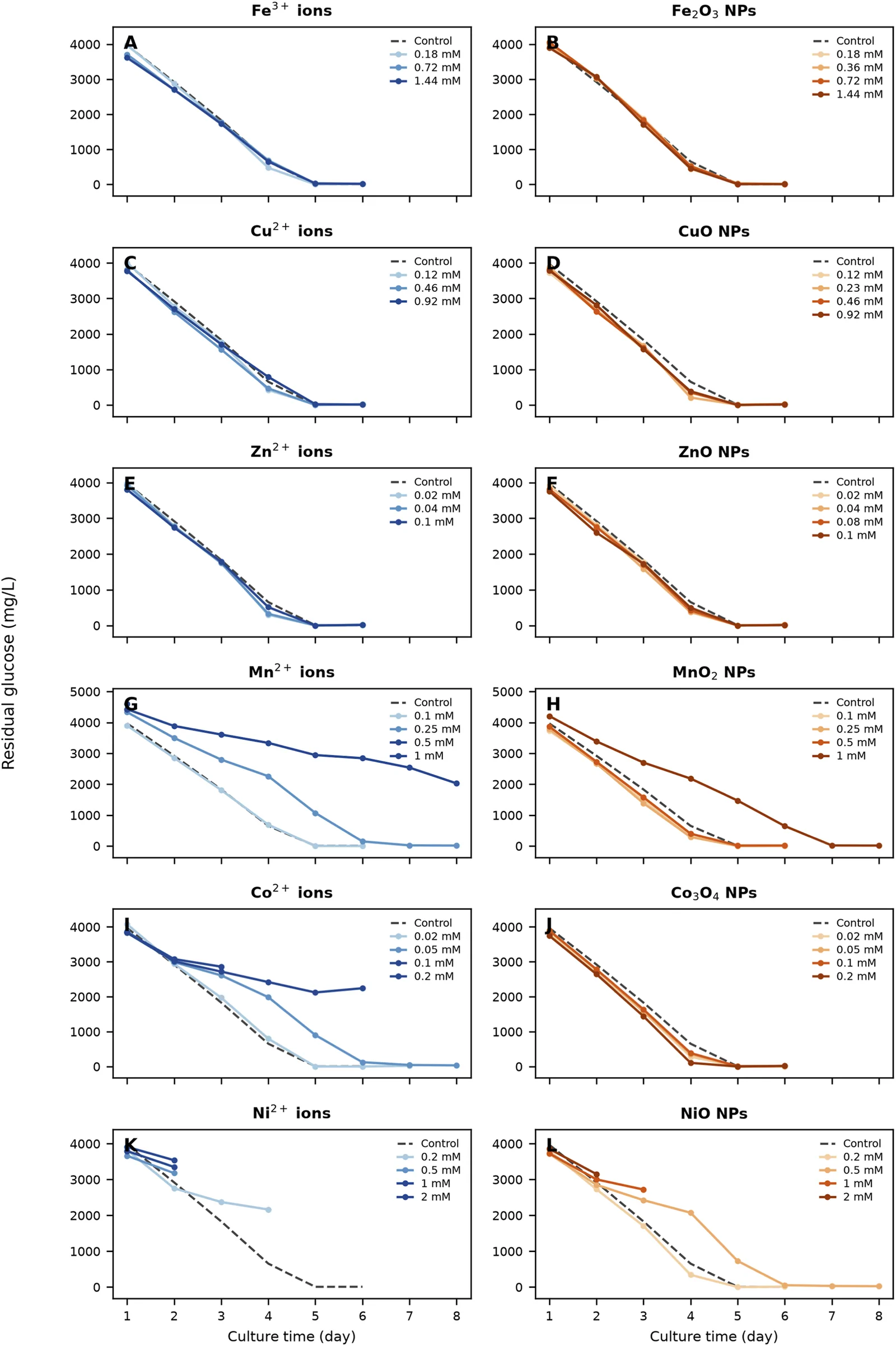
Residual glucose concentration profiles over incubation time for cells cultured with serial concentrations of metal ions and metal oxide nanoparticles. **(A,B)** Fe^3+^ and Fe_2_O_3_ NPs; **(C,D)** Cu^2+^ and CuO NPs; **(E,F)** Zn^2+^ and ZnO NPs; **(G,H)** Mn^2+^ and MnO_2_ NPs; **(I,J)** Co^2+^ and Co_3_O_4_ NPs; **(K,L)** Ni^2+^ and NiO NPs.

**FIGURE 4:**
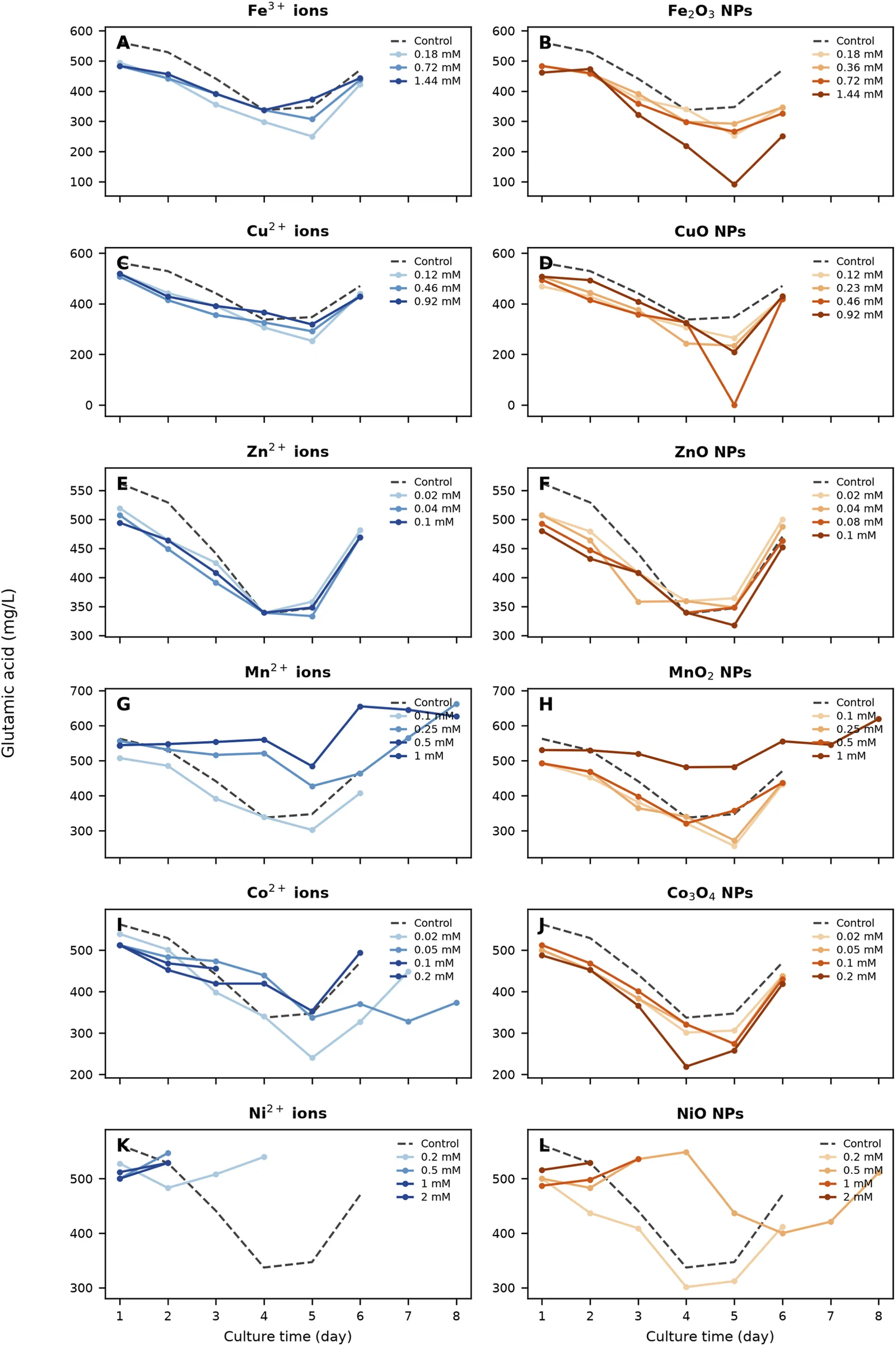
Dynamic glutamic acid concentration curves over incubation time for cells exposed to serially diluted metal ions and metal oxide nanoparticles. **(A,B)** Fe^3+^ and Fe_2_O_3_ NPs; **(C,D)** Cu^2+^ and CuO NPs; **(E,F)** Zn^2+^ and ZnO NPs; **(G,H)** Mn^2+^ and MnO_2_ NPs; **(I,J)** Co^2+^ and Co_3_O_4_ NPs; **(K,L)** Ni^2+^ and NiO NPs.

**FIGURE 5:**
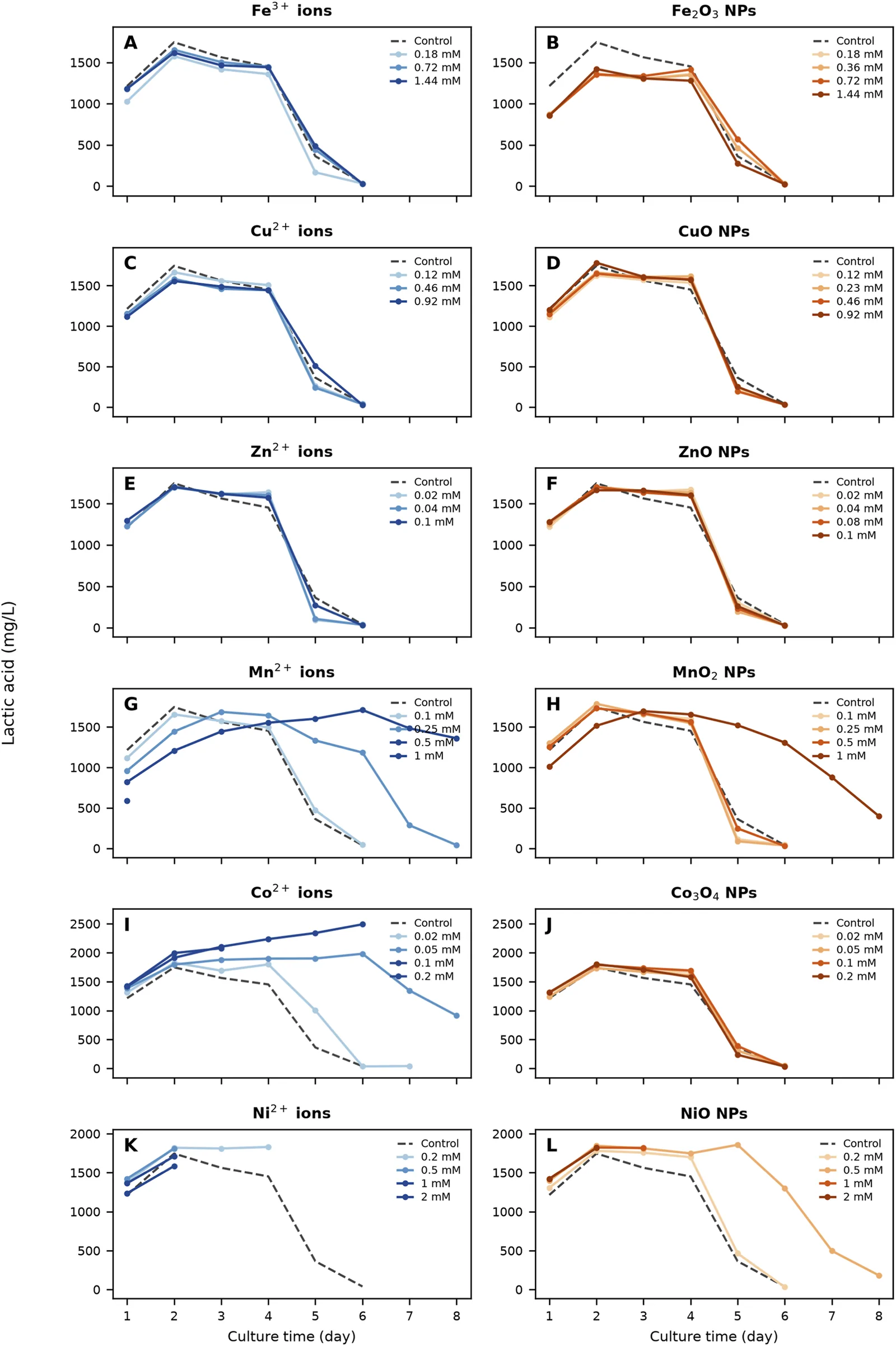
Dynamic lactic acid concentration curves over incubation time for cells exposed to serially diluted metal ions and metal oxide nanoparticles. **(A,B)** Fe^3+^ and Fe_2_O_3_ NPs; **(C,D)** Cu^2+^ and CuO NPs; **(E,F)** Zn^2+^ and ZnO NPs; **(G,H)** Mn^2+^ and MnO_2_ NPs; **(I,J)** Co^2+^ and Co_3_O_4_ NPs; **(K,L)** Ni^2+^ and NiO NPs.

### Microstructural characterization of MnFe-LDH nanoparticles

TEM observation at low magnification indicates that the as-prepared Fe-Mn layered double hydroxides (MnFe-LDH) mainly exhibit two-dimensional ultrathin sheet-like morphology (Figure 6A). The lateral size of nanosheets is mostly distributed in the range of 5–20 nm, and the sheets interlace and stack to form loose aggregates. No discrete impurity particles of metal oxides are obviously observed in the field of view. HRTEM images acquired at different magnifications (200,000 × , 600,000 × , 800,000 × and 1,500,000 ×) show relatively continuous periodic lattice fringes over the sample area, which suggests that the product possesses moderate crystallinity. Interfaces between lattice fringes with diverse orientations can be seen, corresponding to different crystal plane regions of the nanosheets. No large amorphous regions or lattice patterns belonging to other phases are clearly detected, which preliminarily demonstrates that layered double hydroxides containing Mn^2+^ and Fe^3+^ can be obtained under the present synthetic conditions. The XRD pattern demonstrated the characteristic basal reflections of a hydrotalcite-like layered structure at 2θ = 27.4°, 31.76°, 45.48°, and 66.25°, and the corresponding crystal plane indices were (006), (009), (018), and (110), respectively (Figure 6B). Hydrodynamic size of MnFe-LDH was measured by DLS, and the volume-based size distribution indicated the nanosheets form loosely stacked ∼100 nm aggregates without micron-scale agglomeration (Additional Supplementary Figure S1).

**FIGURE 6:**
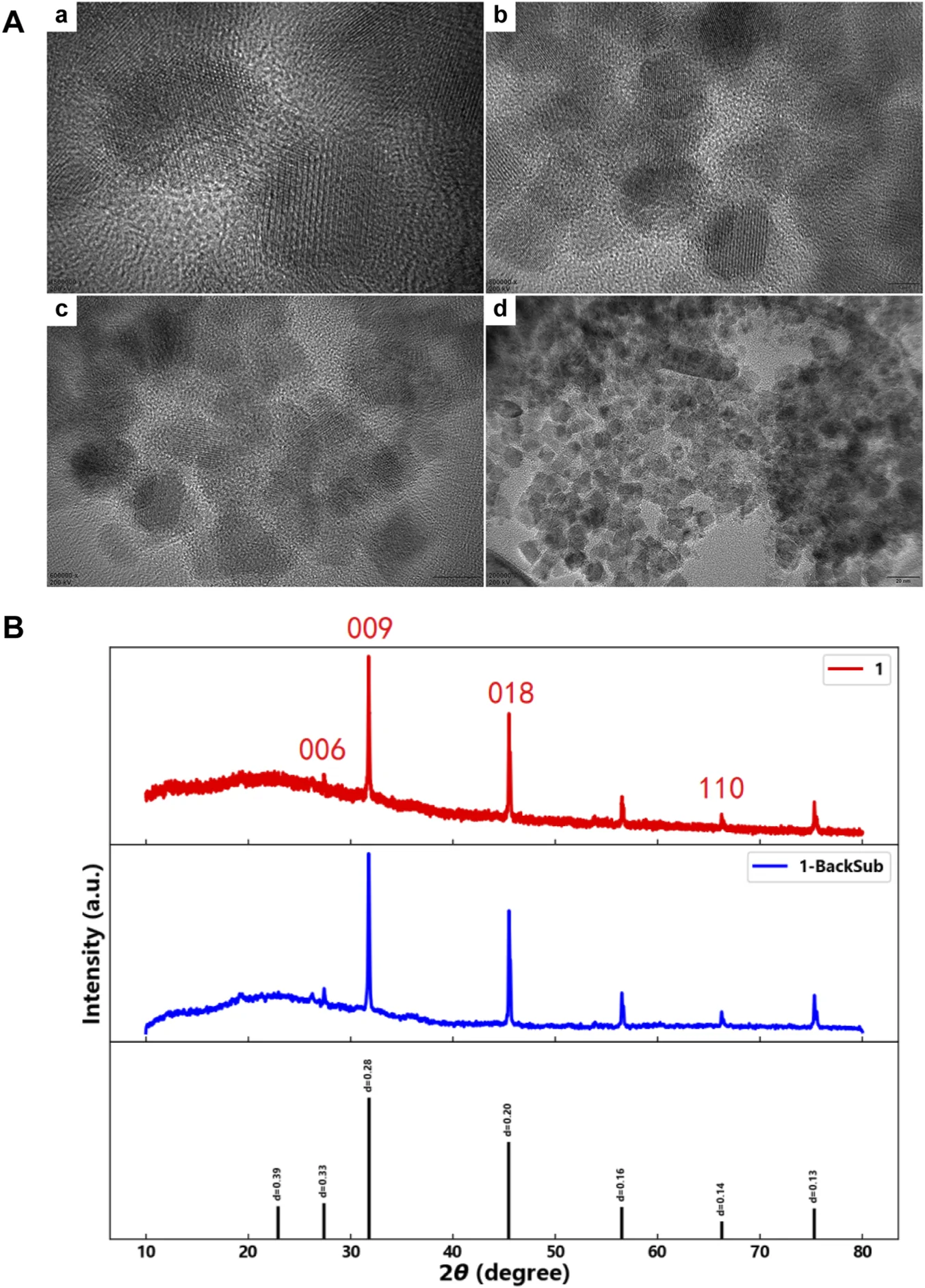
**(A)** Transmission electron microscopy (TEM) characterization of Fe-Mn layered double hydroxides (MnFe-LDH) at different magnifications. **(a)** 1,500,000 × , scale bar = 2 nm; **(b)** 800,000 × , scale bar = 5 nm; **(c)** 600,000 × , scale bar = 10 nm; **(d)** 200,000 × , scale bar = 20 nm. **(B)** XRD patterns of MnFe-LDH.

### Combined Mn^2+^+Fe^3+^ and MnFe-LDH treatments improve cell performance, protein productivity and N-glycan quality over long-term batch culture

Based on the single-metal screening outcomes, four concentration gradients of Mn^2+^+Fe^3+^ ion mixtures and MnFe-LDH nanoparticle composites were applied to a 14-day extended cell culture to evaluate combinatorial effects on cell growth, metabolism, target protein yield and N-glycosylation profiles (Figure 7). Consistent with single-metal observations, low-to-medium gradients (A–C) of both combined treatments sustained higher peak VCD and prolonged viable culture duration compared to the Control group, while the highest D gradient triggered late-stage viability collapse (Figures 7A,B). Long-term metabolic monitoring recapitulated the carbon metabolite trends observed in short-term single-metal cultures: moderate combined metal treatments accelerated glucose consumption, maintained balanced lactic acid turnover and stabilized glutamic acid pools, whereas the D gradient disrupted metabolic homeostasis and led to abnormal metabolite accumulation or depletion (Figures 7C–E). Total secreted recombinant protein was quantified on day 12, 13 and 14, representing the late production phase of the culture (Figure 7F). All A–C gradients of Mn^2+^+Fe^3+^ and MnFe-LDH significantly elevated total protein concentration relative to the untreated Control, with the C gradient yielding the maximum protein titer at all three sampling time points. N-glycan profiling at day 13 further demonstrated that moderate combined metal treatments remodeled glycan subtype distribution; the C gradient notably increased the proportion of complex-type, fully galactosylated glycans and reduced high-mannose glycan fractions, indicating improved glycosylation quality (Figure 7G). These data demonstrate that optimized combined Fe/Mn treatments simultaneously enhance cell growth, protein production and glycan homogeneity in prolonged cell culture.

**FIGURE 7:**
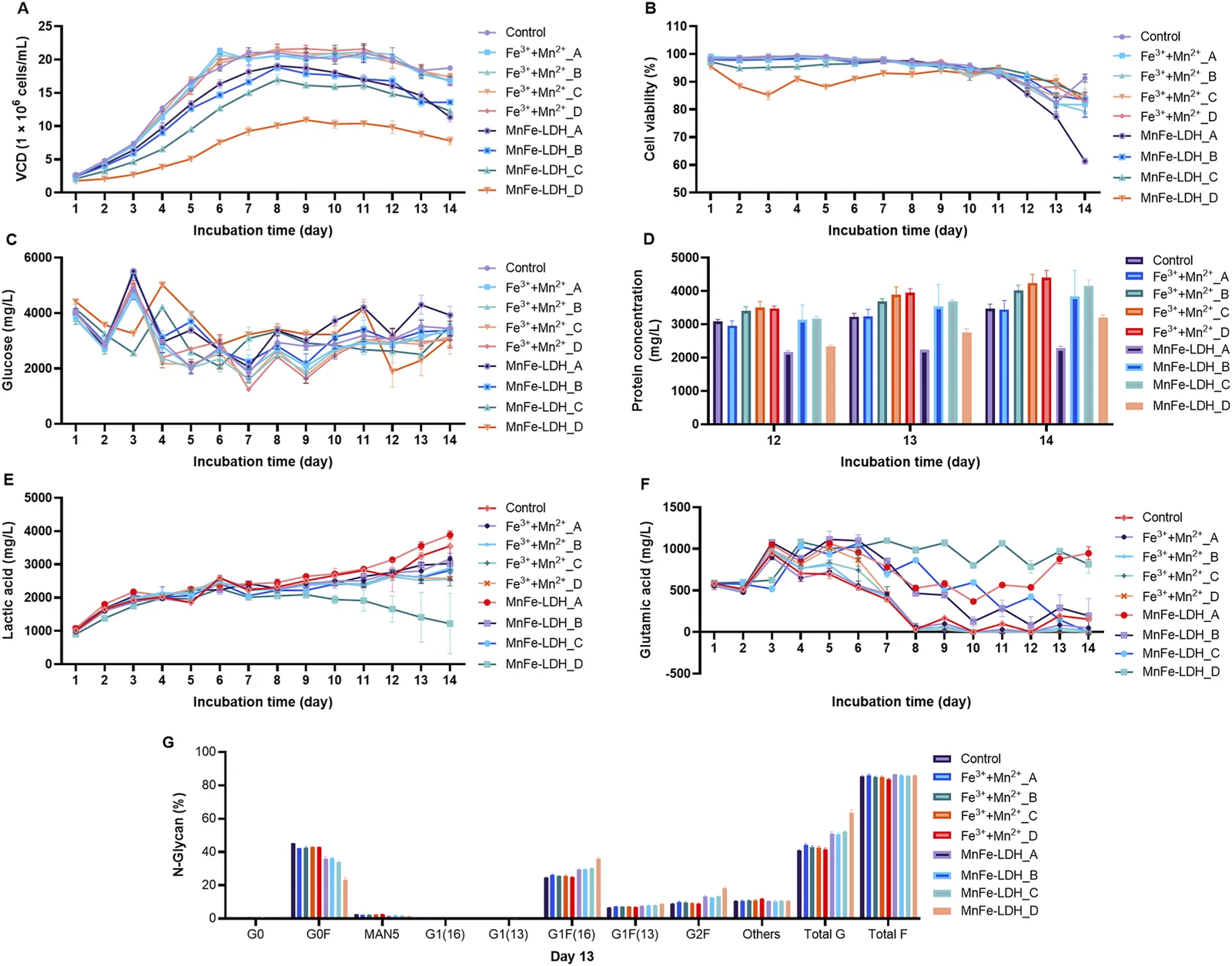
Cell growth, metabolic profiles, total protein production and N-glycan distribution under four gradient treatments of Mn^2+^+Fe^3+^, MnFe-LDH and untreated Control group over a 14-day culture period. **(A)** Time-series viable cell density (VCD, ×10^6^ cells/mL); **(B)** Dynamic cell viability (%); **(C)** Residual glucose concentration (mg/L) in culture medium; **(D)** Medium glutamic acid concentration (mg/L); **(E)** Medium lactic acid concentration (mg/L); **(F)** Total secreted protein concentration (mg/L) quantified on day 12, 13 and 14; **(G)** Relative percentage of distinct N-glycan subtypes determined at day 13.

### Transcriptomic profiling reveals widespread differential gene expression under combined Fe/Mn treatments

To dissect the molecular mechanisms underlying the physiological and phenotypic changes induced by combined Fe/Mn exposure, we performed transcriptome sequencing for all four gradients of Mn^2+^+Fe^3+^ and MnFe-LDH relative to the Control group. Volcano plots visualized the magnitude and statistical significance of differentially expressed genes (DEGs) across all eight treatment conditions (Figure 8). The number of DEGs exhibited a clear dose-dependent increase with rising metal concentration: the mild A gradient induced only hundreds of significant transcriptional changes, while the highest D gradient triggered thousands of upregulated and downregulated genes, highlighting progressive transcriptional remodeling in response to escalating metal stress. Across all groups, the majority of DEGs were significantly upregulated, suggesting broad activation of stress-responsive and metabolic regulatory transcription programs upon Fe/Mn co-exposure.

**FIGURE 8:**
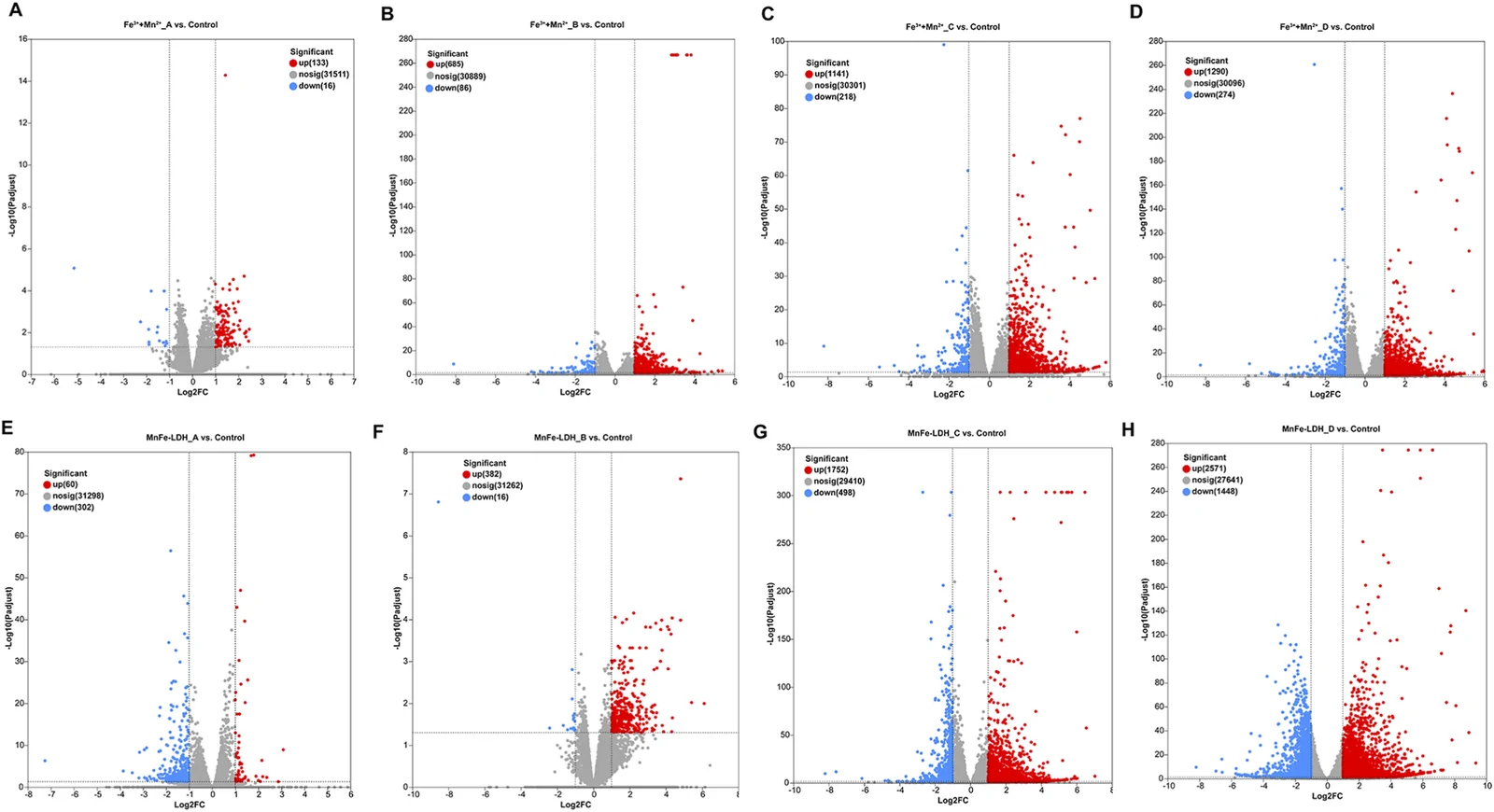
Volcano plots illustrating differentially expressed genes (DEGs) for each treatment group compared with the untreated Control group. **(A–D)** Four gradient concentrations of Mn^2+^+Fe^3+^; **(E–H)** Four gradient concentrations of MnFe-LDH. Red dots represent significantly upregulated genes, blue dots represent significantly downregulated genes, and gray dots denote genes with non-significant expression changes. The x-axis shows log_2_ (fold change), and the y-axis displays -log_10_ (adjusted P-value).

Then, all DEGs were assigned to Cluster of Orthologous Groups (COG) functional categories to categorize core biological processes perturbed by combined Fe/Mn treatments (Figure 9). At low gradients (A, B), DEGs were predominantly enriched in categories associated with energy production/conversion, inorganic ion transport and metabolism, and carbohydrate transport and metabolism, consistent with the observed metabolic shifts in culture supernatants. With increasing metal concentration (C, D), DEG counts expanded sharply in functional groups related to transcription, signal transduction mechanisms, post-translational modification and protein turnover. Notably, genes involved in mitochondrial function, ion channel transport and oxidative stress defense constituted the dominant enriched categories in high-dose groups, indicating severe disruption of ion homeostasis and mitochondrial physiology under elevated Fe/Mn co-stress.

**FIGURE 9:**
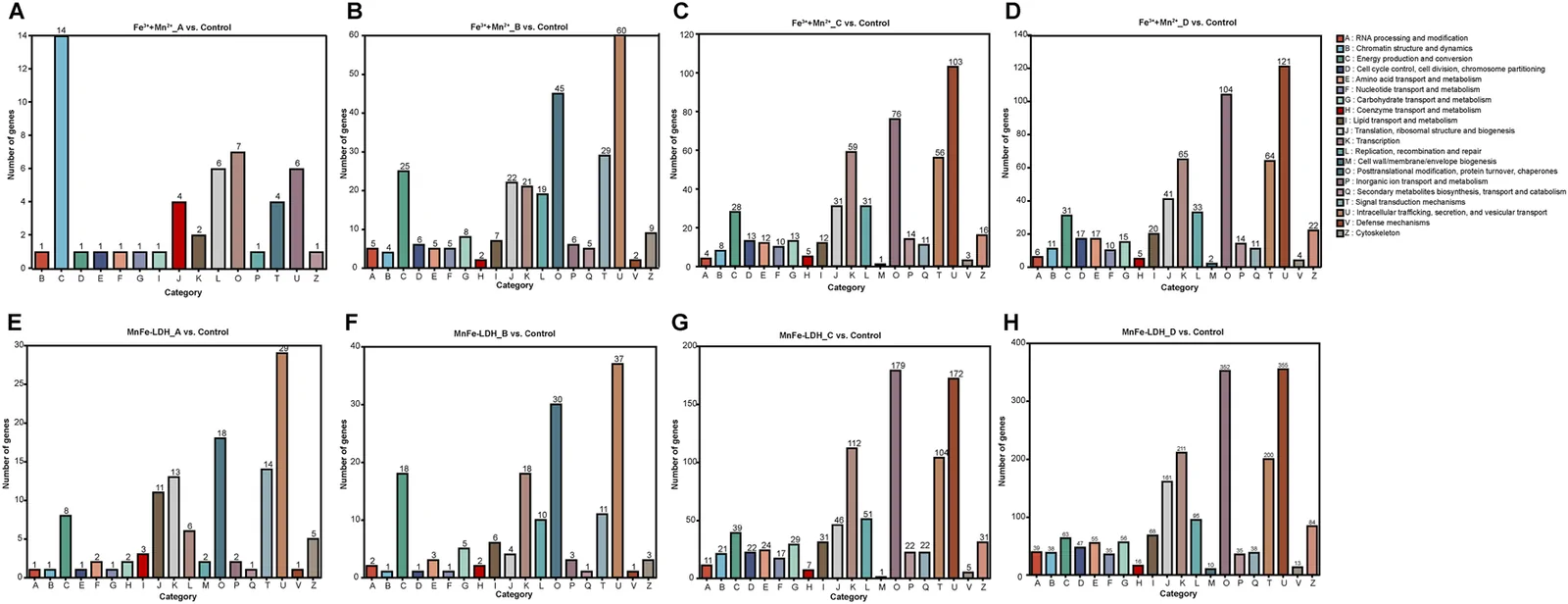
COG functional classification bar charts of differentially expressed genes from each treatment group compared with the Control group. **(A–D)** Four gradient concentrations of Mn^2+^+Fe^3+^; **(E–H)** Four gradient concentrations of MnFe-LDH. The x-axis represents COG functional categories labeled A–Z, and the y-axis indicates the number of assigned differentially expressed genes in each category. The legend on the right defines the biological function corresponding to each category code.

Gene Ontology (GO) bubble enrichment analysis further pinpointed the top perturbed biological processes, cellular components and molecular functions among DEGs (Figure 10). Across all treatment gradients, the most significantly enriched GO terms centered on mitochondrial respiratory chain assembly, oxidative phosphorylation, electron transfer activity, and metal ion transmembrane transport. Low-concentration treatments enriched GO terms linked to basal energy metabolism and ion homeostasis maintenance, while high-dose groups exhibited dominant enrichment in cellular stress response, reactive oxygen species detoxification and negative regulation of mitochondrial function. These transcriptomic enrichment results strongly implicate mitochondrial respiratory machinery as the primary molecular target of combined Fe/Mn exposure.

**FIGURE 10:**
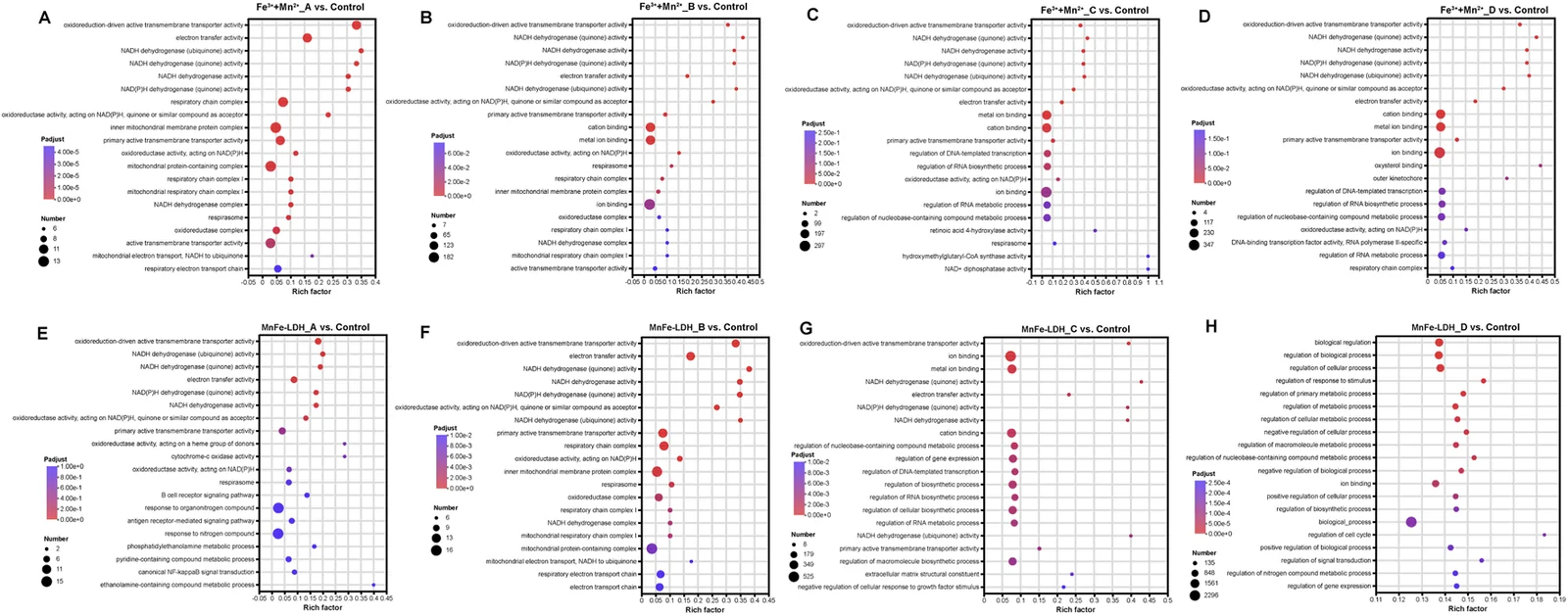
Bubble diagrams of GO enrichment analysis for differentially expressed genes in each treatment versus Control group. **(A–D)** Four gradient concentrations of Mn^2+^+Fe^3+^; **(E–H)** Four gradient concentrations of MnFe-LDH. The x-axis represents enrichment Rich factor, the y-axis displays enriched GO functional terms. The color gradient of bubbles corresponds to adjusted P-value (Padj), and bubble size indicates the number of enriched differential genes.

### Combined Fe/Mn treatments dose-dependently alter the expression of key mitochondrial respiratory chain genes

Given the consistent enrichment of mitochondrial respiration-related terms in transcriptomic analyses, we extracted the transcript levels of six core mitochondrial respiratory chain genes (COX2, COX3, ATP6, ND3, ND4 and ND4L) together with B4GALT1, which encodes the galactosylation enzyme β-1,4-galactosyltransferase 1, from the same RNA-sequencing dataset as a targeted within-dataset comparison (Figure 11). One-way ANOVA with Tukey’s multiple comparison test confirmed significant intergroup differences (p < 0.05), annotated by distinct lowercase letter labels on each bar. Compared with the untreated Control, all Mn^2+^+Fe^3+^ and MnFe-LDH gradients significantly upregulated the mRNA abundance of the six mitochondrial genes in a dose-dependent manner; expression levels increased sequentially from gradient A to D. The highest MnFe-LDH_D group displayed the maximum log_10_ (TPM+1) expression value for every tested respiratory gene, with statistically significant elevation relative to all other treatment groups. Intermediate gradients (B, C) exhibited moderate upregulation, matching the balanced metabolic and growth performance observed in long-term batch cultures.

**FIGURE 11:**
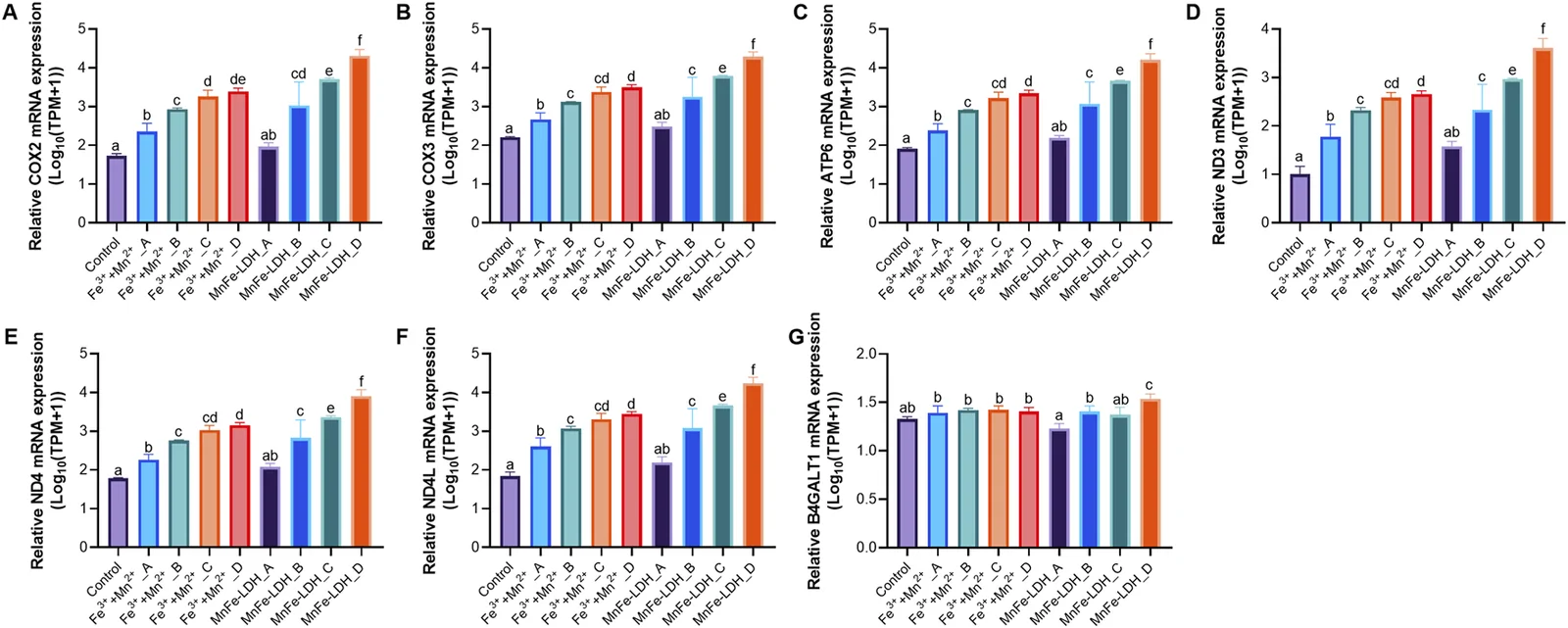
Bar charts showing relative mRNA expression levels of six mitochondrial respiratory chain genes under Control, four gradient Mn^2+^+Fe^3+^ treatments and four gradient MnFe-LDH treatments. **(A)** COX2; **(B)** COX3; **(C)** ATP6; **(D)** ND3; **(E)** ND4; **(F)** ND4L; **(G)** B4GALT1. mRNA expression values were transformed as log_10_ (TPM+1). One-way ANOVA with Tukey’s multiple-comparison test was performed across all experimental groups simultaneously. Data are presented as mean ± standard deviation; bars labeled with distinct lowercase letters **(A–G)** differ significantly (p < 0.05), whereas bars sharing identical letters do not differ significantly.

## Discussion

This study provides a comprehensive evaluation of six transition metal ions and their corresponding oxide nanoparticles on CHO cell culture performance. Our results demonstrate that low concentrations of Fe^3+^ and Mn^2+^ promote cell proliferation, while high concentrations of Cu^2+^, Zn^2+^, Co^2+^, and Ni^2+^ significantly inhibit growth and reduce viability. These findings align with the well-established dual role of transition metals as both essential cofactors and potential toxicants (Jomova et al., 2022; Valko et al., 2005). The dose-dependent hormetic response observed for Fe and Mn is consistent with previous reports demonstrating that these metals support mitochondrial function and enzymatic activities at physiological concentrations but induce oxidative stress at supraphysiological levels (Chen et al., 2015; Maio and Rouault, 2015).

A key observation in this study is that metal oxide nanoparticles consistently exhibited lower cytotoxicity compared to their ionic forms at equivalent metal concentrations. We propose that this attenuation reflects the slower dissolution kinetics of nanoparticles, which would be expected to deliver metal ions more gradually into the culture medium (Xia et al., 2008). However, no time-dependent dissolution or ion-release measurements were performed under the present culture conditions, so this explanation remains an untested hypothesis rather than a demonstrated mechanism for the materials studied here. Specifically, Wang et al. demonstrated that CuO nanoparticles undergo profound dissolution at lysosomal pH (4–5) via ligand-assisted ion release, where amino acid complexation is a major contributor, whereas dissolution is negligible at neutral pH (Wang et al., 2013). This pH-dependent dissolution mechanism has been proposed to explain why nanoparticles, once endocytosed into acidic endolysosomal compartments (Behzadi et al., 2017; Stern et al., 2012), would release metal ions gradually and thereby attenuate acute cytotoxic effects. In CHO-K1 cells specifically, Hanot et al. showed that iron-oxide nanoparticle toxicity profiles correlate strongly with their cellular uptake kinetics, and that surface PEGylation reduces both uptake and cytotoxicity (Hanot et al., 2015). Our finding that Fe_2_O_3_ nanoparticles showed markedly lower toxicity than Fe^3+^ ions is consistent with the concept that the nanoparticle form limits the bioavailability of free Fe^3+^ that would otherwise catalyze Fenton reactions at the cell surface (Winterbourn, 2008; Nowak-Jary and Machnicka, 2024). Similarly, CuO and ZnO nanoparticles exhibited attenuated toxicity compared to Cu^2+^ and Zn^2+^ ions, consistent with the solubility-dependent toxicity model proposed by (Wang et al. (2013) and the intracellular solubility paradigm established by Studer et al. (2010). Importantly, the attenuated cytotoxicity of nanoparticles should not be attributed to the particle form alone; because the biological effects of metal oxide nanoparticles are largely mediated by intracellular dissolution and ion release (Xia et al., 2008; Studer et al., 2010), the differences observed between ionic and nanoparticulate supplementation primarily reflect differences in ion delivery kinetics rather than fundamentally distinct toxic mechanisms, and dedicated particle-only controls would be required to fully separate these two contributions. Nowak-Jary et al. further catalogued the oxidative stress mechanisms induced by magnetic iron oxide nanoparticles, including mitochondrial dysfunction, glutathione depletion, and NF-κB-mediated inflammatory pathways, and noted that surface coating and particle size significantly modulate ROS generation (Nowak-Jary and Machnicka, 2024). In CHO-K1 cells, ZnO nanoparticles have been shown to induce cell polarisation linked to cytoskeleton ablation through proteomic alterations in Rac-Rho signaling pathways (Yadav et al., 2021), and metal-based nanoparticles more broadly trigger cytotoxicity cascades centered on oxidative stress (Min et al., 2023), while BSA protein corona formation on ZnO nanoparticles reduces both cytotoxicity and ROS generation, although conformational changes in adsorbed protein can partially compromise this protective effect (Žūkienė and Snitka, 2015). This protein corona effect is particularly relevant to bioprocessing applications, where culture medium proteins inevitably coat nanoparticle surfaces. Schardosim et al. reported that metal oxide nanoparticles can induce DNA damage and chromosome instability in CHO-K1 cells even at moderate concentrations, with crystalline forms showing greater genotoxicity than amorphous counterparts (Schardosim et al., 2022), underscoring the importance of crystal phase in addition to surface chemistry and size.

Transcriptomic analysis identified mitochondrial respiratory chain genes as the primary molecular targets of metal ion perturbation. Iron-sulfur (Fe-S) clusters are essential cofactors for multiple subunits of complexes I, II, and III, and their biogenesis depends on the mitochondrial ISC assembly machinery (Maio and Rouault, 2015; Lill, 2009). Our observation that high Fe^3+^ concentrations paradoxically impaired mitochondrial gene expression likely reflects disruption of Fe-S cluster biogenesis through iron overload, which can promote Fenton chemistry and oxidative damage to Fe-S cluster-containing proteins (Winterbourn, 2008; Jiang et al., 2021). Conversely, Mn^2+^ supplementation at optimal concentrations enhanced expression of mitochondrial complex I and II genes, consistent with the role of Mn as a cofactor for MnSOD (SOD2), the primary mitochondrial antioxidant enzyme (Azadmanesh and Borgstahl, 2018; Holley et al., 2011). Our finding that Mn supplementation partially rescued Cu-induced ROS elevation supports the protective role of MnSOD in mitigating metal-catalyzed oxidative damage (Azadmanesh and Borgstahl, 2018; Holley et al., 2011). The synergistic effect of combined Mn^2+^ and Fe^3+^ supplementation on CHO cell growth and N-glycosylation represents a particularly significant finding. Mechanistically, this synergy likely reflects complementary roles: Fe supports mitochondrial respiration and ATP production through Fe-S cluster-containing complexes, while Mn serves as a cofactor for both SOD2 (mitigating Fe-catalyzed ROS generation) and B4GALT1 (enhancing terminal galactosylation) (Ramakrishnan and Qasba, 2002; Gramer et al., 2011; Williamson et al., 2018). The protective effect of Mn against Fe-induced oxidative stress through SOD2-mediated superoxide scavenging reduces Fenton reaction-mediated hydroxyl radical generation, thereby preserving mitochondrial integrity (Winterbourn, 2008; Jiang et al., 2021).

Our findings on metal-mediated regulation of N-glycosylation have significant implications for biopharmaceutical manufacturing. The enhancement of terminal galactosylation by Mn^2+^ supplementation is consistent with the established role of Mn as an essential cofactor for B4GALT1 (Gramer et al., 2011; Williamson et al., 2018). Recent studies have demonstrated that temporal galactose-manganese feeding in fed-batch and perfusion bioreactors modulates UDP-galactose pools, resulting in more homogeneous glycoform distributions (Gyorgypal et al., 2025; Sha and Yoon, 2019). Our results extend these findings by showing that co-supplementation of Fe with Mn further enhances galactosylation, likely through increased ATP availability from improved mitochondrial respiration, which drives nucleotide sugar biosynthesis (Jimenez del Val et al., 2011; Gyorgypal et al., 2025). Conversely, high concentrations of Cu^2+^ and Zn^2+^ reduced galactosylation, consistent with reports that metal impurities in culture media can adversely affect glycosylation patterns (Zhang et al., 2016). Whether Fe^3+^ cooperates with Mn^2+^ beyond sustaining respiration- and ATP-driven nucleotide sugar biosynthesis, and whether glycosylation-enzyme transcripts themselves are altered in our differentially expressed gene dataset, remains to be clarified; systematic reexamination of glycosylation-enzyme transcripts and dedicated isotope-tracing experiments are warranted. We note, however, that Mn^2+^ acts directly as the B4GALT1 cofactor, providing a post-translational route for enhanced galactosylation (Ramakrishnan and Qasba, 2002; Williamson et al., 2018).

From a practical perspective, our findings have significant implications for CHO cell culture media optimization in biopharmaceutical manufacturing. The identification of optimal concentration ranges and timing strategies for Mn and Fe supplementation provides a rational basis for designing feeding strategies that simultaneously enhance cell growth and glycosylation quality (Gyorgypal et al., 2025; Polanco et al., 2023). The potential of nanoparticle-mediated metal delivery to attenuate cytotoxicity while maintaining bioavailability warrants further investigation in large-scale bioreactor systems (Hanot et al., 2015; Chusuei et al., 2013). As highlighted by Nowak-Jary et al., the surface properties of iron oxide nanoparticles critically determine their biological interactions, suggesting that rational surface engineering could further improve biocompatibility in cell culture applications (Nowak-Jary and Machnicka, 2024). Future studies should focus on elucidating the precise mechanisms of nanoparticle uptake and intracellular dissolution in CHO cells using live-cell imaging, investigating the interplay between metal supplementation and nucleotide sugar metabolism through metabolomic approaches, and developing predictive models that integrate transcriptomic and metabolomic data to optimize metal feeding strategies in real time (Reddy et al., 2023). The convergence of advanced omics technologies, machine learning, and nanotechnology holds promise for achieving rational, knowledge-driven control of glycosylation in biopharmaceutical manufacturing. Additionally, the influence of nanoparticle surface properties on membrane integrity and intracellular signaling, such as the ZnO NP-induced cholesterol disruption reported by Kuebodeaux et al. (Kuebodeaux et al., 2018), warrants systematic investigation in CHO bioprocessing contexts to ensure that nanoparticle-based strategies do not inadvertently compromise membrane-dependent quality attributes.

Several limitations of this study should be acknowledged. First, although N-glycan profiling demonstrated improved galactosylation under combined Mn/Fe supplementation, glycans were analyzed from culture supernatant at a single time point (day 13) and at the released N-glycan level; confirmation at the purified product protein level, including site-specific glycopeptide analysis and temporal monitoring of glycoform dynamics, will be required to establish product quality at the protein level. The MnFe-LDH material was characterized by TEM imaging, powder XRD and DLS, which establish sheet-like morphology, a crystalline cubic-type lattice and a unimodal hydrodynamic size distribution of approximately 100 nm in aqueous dispersion; zeta potential, XPS-type surface-composition analysis, characterization in complete culture medium and time-dependent ion-release/dissolution kinetics under culture conditions were not determined, so the relative contributions of released ions and particle-specific effects cannot be fully separated at present and the proposed slower-release mechanism remains untested. Third, the fate of residual metal ions and nanoparticles during harvest and downstream purification was not assessed; potential metal or nanoparticle carryover into the final product, and its implications for product quality, regulatory compliance and process economics, should be evaluated before nanoparticle-based strategies are adopted at manufacturing scale. Finally, all experiments were conducted in small-scale shake-flask batch culture, and extrapolation to chemically defined media, fed-batch or perfusion operation, and industrially relevant bioreactor conditions will require systematic validation.

## Data Availability

The raw data supporting the conclusions of this article will be made available by the authors, without undue reservation.

## Supplementary material

SUPPLEMENTARY FIGURE S1 Hydrodynamic size distribution of the bilayer Mn-Fe nanocomposite (MnFe-LDH) nanoparticles determined by dynamic light scattering (DLS). **(A–C)** Volume-weighted size distributions recorded in three replicate measurements (Sample 1, Sample 2 and Sample 3) on a Malvern Zetasizer Nano ZS90.
